# *In-vivo* MRI Reveals Changes to Intracerebral Vasculature Caliber in HIV Infection

**DOI:** 10.3389/fneur.2019.00687

**Published:** 2019-06-26

**Authors:** Paba M. De Alwis, Bryan R. Smith, Tianxia Wu, Cristah Artrip, Sally Steinbach, Caryn Morse, Chuen-Yen Lau, Stanley I. Rapoport, Joseph Snow, Edmund Tramont, Daniel S. Reich, Govind Nair, Avindra Nath

**Affiliations:** ^1^National Institute of Neurological Disorders and Stroke, National Institutes of Health, Bethesda, MD, United States; ^2^Clinical Center, National Institutes of Health, Bethesda, MD, United States; ^3^National Institute of Allergy and Infectious Diseases, National Institutes of Health, Bethesda, MD, United States; ^4^National Institute on Alcohol Abuse and Alcoholism, National Institutes of Health, Bethesda, MD, United States; ^5^National Institute of Mental Health, National Institutes of Health, Bethesda, MD, United States

**Keywords:** HIV, vasculitis, MRI, intracranial vessel caliber, cerebrovascular disease

## Abstract

**Objective:** To characterize cerebral arterial remodeling in HIV-infected (HIV+) individuals *in-vivo*, and to study its clinical and immunological associations.

**Methods:** T2^*^-weighted magnetic resonance imagining sequences was used to determine cross-sectional area (vascular caliber) of the anterior (A1 segment) and middle (M1 segment) cerebral arteries in HIV- (control) and HIV+ subjects on antiretroviral therapy. Correlations of A1 caliber with clinical, demographic parameters, and immunological markers in cerebrospinal fluid (CSF) were determined using multivariable analyses.

**Results:** A1 and M1 calibers from 22 HIV- control subjects (age: median 48.5 years, range 22-60 years, 55% male) and 61 HIV+ subjects (age: median 53 years, range 25–60 years, 67% male) were studied. ANCOVA, adjusting for ethnicity and sex (age was not correlated with M1 or A1 caliber in either group), revealed that HIV+ subjects had larger caliber in the A1 segment than HIV- subjects (4.95 ± 0.14 mm^2^, and 4.47 ± 0.21 mm^2^ respectively, *p* = 0.048), but caliber of the M1 segment did not differ among the groups (7.21 ± 0.14 mm^2^ and 7.09 ± 0.23 mm^2^ respectively, *p* = 0.65). In the HIV+ cohort, longer disease duration and higher current CD4 T-cell count were associated with reduced A1 caliber (*r* =−0.42 and −0.33 respectively, *p* < 0.05). In addition, increase in cardiovascular disease risk (CVD risk) was associated with a decrease in A1 caliber in the HIV group (*r* = −0.35, *p* < 0.05).

**Conclusions:** This cross-sectional study reveals an increase in A1 caliber in the HIV+ cohort, compared to control subjects, which is especially prominent in early phase of the disease. This increase in caliber may be associated with acute pathological processes in HIV during the initial stages of infection resulting in loss of compliance or thinning of the arterial wall. At later stages, such changes may be confounded by arteriosclerotic changes that are common in later stages of HIV infection. This study suggests there is extensive vessel remodeling in various stages of infection. Long-term longitudinal follow-up of this cohort is planned to further verify this hypothesis and to better understand this MRI marker of intracranial vascular caliber.

## Introduction

Despite the effectiveness of antiretroviral drugs, it is becoming increasingly clear that vascular compromise frequently accompanies HIV disease (1–5). Autopsy studies in the combination antiretroviral therapy (cART) era have found increased vascular caliber in patients with HIV infection (2–5). Such studies have also reported remodeling of the microvasculature, which may reflect structural damage from inflammation and/or immune dysfunction (1, 6). For instance, pathology studies have indicated that there is thinning of the medial arterial layer in people with HIV infection (2). In addition to such smooth muscle layer changes, damage in the innermost endothelial layer in blood vessels as seen in HIV could also impact vessel compliance and structure (7). The effects of such changes and the resulting vascular remodeling may have wide-ranging implications in these HIV patients, such as increased risk of cerebrovascular disease, which occurs with higher prevalence in both untreated and successfully treated HIV patients compared to the general population (8, 9). A technique to study such vascular remodeling *in-vivo* could therefore be very useful in assessing patient outcomes.

Despite its potential importance, there are only a few techniques that allow reliable determination of intracranial vascular caliber *in-vivo*. Most vascular studies have been performed postmortem on fixed brain tissue samples (2–5) where the *ex-vivo* measurements may be affected by alterations to brain structures during fixation and sectioning. Magnetic resonance imaging (MRI) confers the unique opportunity to measure intracranial vessel caliber at high resolution *in-vivo*. Additionally, such measurements can be performed longitudinally through the course of the disease. Here, we examined cerebrovascular caliber in a cross-sectional study of HIV-seronegative control subjects (HIV-) and HIV-seropositive subjects (HIV+), using high-resolution T2^*^-weighted MRI sequences (10–13) and a novel vessel caliber quantification method developed in-house, which was similar to a previously published method for spinal cord cross-sectional area calculation (14, 15).

## Methods

### Study Participants

The NIAID and CNS Institutional Review Board approved the study protocols (clinicaltrials.gov NCT01875588 and NCT00001248), and all participants provided written informed consent. Individuals with and without HIV infection, between the ages of 18–61 years and living in the Washington DC metropolitan area, were recruited. Additionally, direct comparison of MRI techniques to detect intracranial vasculature was performed on an additional subject (32 y.o. female, Supplementary Figure 1). Subjects were eligible for participation if they met the following inclusion criteria: no history of CNS infection, no concurrent unstable psychiatric illness, no history of traumatic brain injury, and no contraindication to MRI scanning. All HIV+ subjects were required to be on antiretroviral therapy with a plasma HIV RNA <40 copies/mL. HIV- participants were recruited from the same locale to best match the demographic, social, and comorbid conditions, and, to the extent possible, race, age, and education levels, of the HIV+ cohort.

### Demographic and Clinical Variables

Information on duration of HIV infection, nadir CD4+ T-cell count, and illicit drug use were obtained at subject interviews on site and through review of medical records when possible (a self-reported use and/or positive laboratory screen for marijuana, cocaine, crack, heroin, methadone, or hallucinogens was taken as positive evidence of drug use). Laboratory variables, including current CD4+ T-cell count, were measured within 1 week of the MRI. Insulin resistance was calculated with the HOMA-IR method using fasting glucose and insulin levels (16). Cardiovascular disease risk score (CVD risk) was obtained using the American Heart Association guidelines for the assessment of 10-year estimated risk for atherosclerotic CVD, which was calculated using age, sex, race, total cholesterol, HDL cholesterol, diastolic and systolic blood pressure, blood-pressure-lowering medication use, diabetes status, and smoking status.

### MRI Acquisition

All participants completed an MRI scan on a 3T Philips Achieva scanner (Philips Medical Systems, Best, the Netherlands) with an 8-channel head coil. For the purposes of vessel analysis, 3D T2^*^-weighted (hereafter “T2^*^”) images were acquired with a segmented 3D echo-planar-imaging sequence with repetition time 54 ms; echo time 30 ms; 10° flip angle; 15 lines per shot; 2 averages; 4 min 14 s acquisition time; 0.55 mm isotropic resolution, resulting in voxel size of 0.17 mm^3^. The sequence was acquired immediately following intravenous injection of gadobutrol (0.1 mmol/kg). The presence of both MRI contrast agent and endogenous deoxyhemoglobin within the lumen of vessels causes T2^*^ shortening, making the vasculature prominently hypointense in the T2^*^ images. For a direct comparison of intracranial vascular imaging techniques, 3D Time-Of-Flight angiography (repetition time 21 ms; echo time 3.4 ms; 18° flip angle; 5 min acquisition time) and 3D T2^*^ were acquired on a volunteer after injection of 0.1 mmol/kg gadobutrol.

### Vessel Analysis

The A1 segment of the anterior cerebral artery and the M1 segment of the middle cerebral artery were chosen for the analysis because of their characteristic locations on the T2^*^ images and ease of tractability on the analysis software, which allowed reliable detection and accurate characterization of caliber.

**Figure 1:**
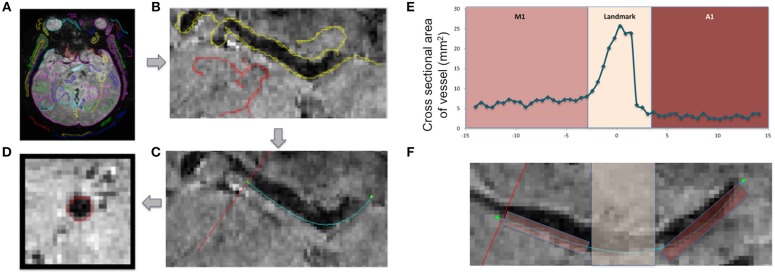
Technique for vessel caliber calculation and visualization. Axial slice depicting the vasculature of interest is identified on a high resolution T2^*^-weighted image **(A)**. The edge of each vessel is identified **(B)** and fit to a polynomial (teal line in **C**). Slices are reconstructed perpendicular to the teal line (red line in **C**) along the length of the vessel, and the cross-sections are visualized and calculated on these reconstructed slices **(D)**. Plots of cross-sectional area **(E)** along the length of a vessel **(F)** are presented in a control subject, showing M1 and A1 segments. The region of increased area at the location where the internal carotid artery merges with Circle of Willis was used as a landmark for aligning patient data (Landmark).

An algorithm written in MATLAB was used to analyze the cross-sectional area of vessels (Figures 1A–F). Contours corresponding to the artery of interest were selected from an axial slice of the patient's T2^*^ image (Figures 1A,B). The edges of the vessel encompassing M1 and A1 segments, including the region where the internal carotid artery meets the Circle of Willis, were fit to a polynomial function (teal line in Figure 1C). Images were reconstructed perpendicular to the polynomial, displaying the arterial segments in cross-section (red line in Figure 1D). The process was repeated along the length of the vessel to generate a cross-sectional area plot for the entire segment (Figures 1E,F). Where the internal carotid artery bifurcates into the M1 and A1 segments there was a large increase in the cross-sectional area, seen as a spike (Figure 1E), and this feature served as a landmark that was used to reliably line up data points across subjects. The median cross-section areas of the left and right hemispheric segments (box in Figure 1F) were averaged to derive mean M1 and A1 calibers for each patient.

### Statistical Analysis

All data reported in this cross-sectional study are mean ± standard deviation unless otherwise mentioned. Reliability of the method was assessed using intraclass correlation coefficient (ICC) by calculating the vessel caliber twice by the same rater. Fisher's exact test (sex, ethnicity, and drug use) and Wilcoxon test (age, current CD4+ T-cell count, CVD risk, and insulin resistance) were used to compare demographic and clinical variables between HIV+ and HIV- cohorts. ANCOVA model was used to examine the cohort difference in M1 and A1 with sex and race considered as covariates. The association of each clinical variable with M1/A1 vessel caliber was evaluated using Pearson's correlation coefficient, and linear regression analysis (M1 or A1 as dependent variable, ethnicity, sex and each clinical variable as independent variables) for HIV+ cohort only. Shaprio-Wilk method was applied to residuals for testing normality assumption above parametric-tests.

## Results

Thirty HIV- subjects and 86 HIV+ subjects on antiretroviral therapy were initially recruited for the study. Of these, only data from participants who were able to get MRI contrast agent (had no contraindications to receiving gadobutrol) and who had T2^*^ images with clear and traceable M1 and/or A1 segments (no image artifacts due to motion or other reasons), was used in the analysis. Consistent with these requirements, data from 22 HIV- subjects and 61 HIV+ subjects were used for the final analysis. Demographic and clinical information of these participants are shown in Table 1. ICC calculated for 17 subjects (age: median 48.5 years, range 30–60 years, 63% male) for M1 was 0.92, with a 95% confidence interval [0.80, 0.97], and for A1 0.83, with a 95% confidence interval [0.58, 0.94], indicating good reliability for the measurement (17).

**Table 1:** Demographic and clinical variables of subjects^*^.

	**HIV- (*n* = 22)**	**HIV+ (*n* = 61)**	***P-*value**
A1 cross-sectional area, least squares mean ± SE, (mm^2^)	4.47 ± 0.21	4.95 ± 0.14	0.0483^a^^*^
M1 cross-sectional area, ls mean ± SE, (mm^2^)	7.09 ± 0.23	7.21 ± 0.14	0.6457^a^^*^
Age, median (range), (years)	48.5 (22–60)	53 (25–60)	0.0227^b^
Duration of HIV infection, mean ± SD (years)	N/A	18.5 ± 8.5	–
Sex, n male (%)	12 (55%)	41 (67%)	0.3110^c^
Ethnicity, *n* African American (%)	15 (68%)	38 (62%)	0.7965^c^
Nadir CD4+ T-cell count, median (range), (cells/mL)	N/A	186 (1-800)	–
Current CD4+ T-cell count, median (range), (cells/mL)	844 (333–1,282)	499 (124–1,536)	<0.0001^2^
CVD risk, median (range), (%)	2.3 (0–10)	5.9 (0–23.6)	0.0091*^b^*
Insulin resistance median (range), HOMA-IR	2 (0.6–10.8)	1.9 (0–13.1)	0.7444^b^
Drug use, n of drug users (%)	6 (27%)	23 (38%)	0.4428^c^

Statistical test used:

a *ANCOVA*,

* Least squares means calculated after controlling for sex and race;

b Wilcoxon test;

c *Fisher's exact test*.

After exclusion of scans that could not be analyzed, HIV+ subjects were older than the HIV- subjects (*p* = 0.0227; Table 1) and had a higher 10-year projected risk of CVD (*p* = 0.0091; Table 1). As expected, HIV+ subjects had lower current CD4+ T-cell counts than HIV- participants (*p* < 0.0001). Prevalence of drug use and distributions of sex, ethnicity, and other clinical variables did not differ between groups. HIV+ subjects had a mean duration of infection of 18.5 years and a mean nadir CD4+ T cell count of 186 cells/mL (Table 1).

**Figure 2:**
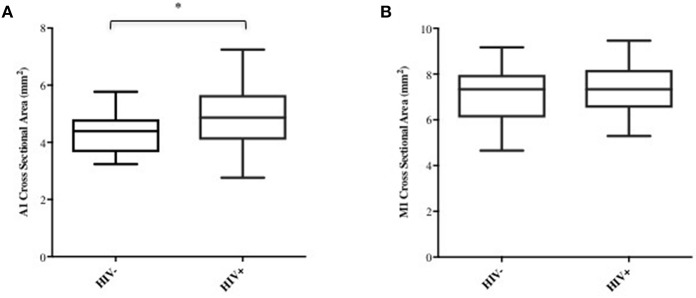
A1 and M1 vessel caliber across cohorts. Group-average plots of vessel caliber in A1 **(A)** and M1 **(B)**. A1 segment shows increased caliber in HIV+ subjects (^*^p < 0.05).

**Figure 3:**
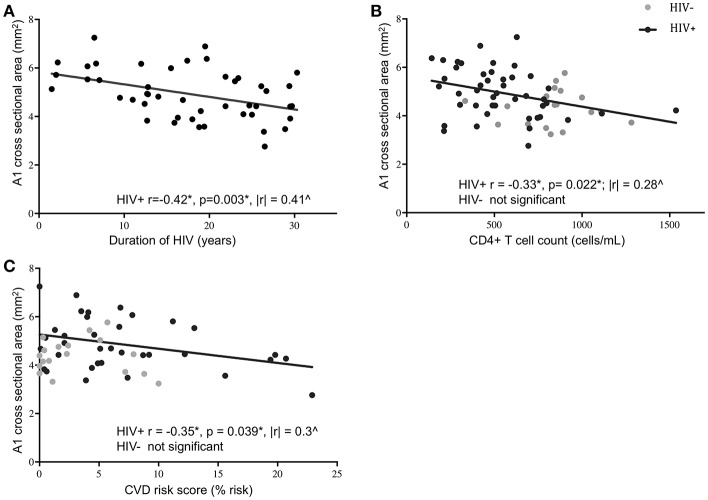
Association of A1 caliber with clinical and demographic variables. A1 vessel caliber showed significant correlation with duration of HIV infection **(A)**, current CD4+ T cell levels **(B)**, and 10-year projected cardiovascular disease risk score **(C)** in HIV+ subjects (black circles) but not in HIV- controls (gray circles). Significant correlations in the HIV+ subjects are indicated by the black line (^*^Pearson's correlation coefficient and *p*-value; ^∧^partial correlation coefficient from linear regression model with ethnicity and sex as covariates).

Age, sex, and race were initially picked as possible covariates to test when assessing differences between cohorts in M1 and A1 caliber. Correlation analysis indicated that age had little association with M1 or A1 (HIV-: *r* = −0.02 for M1 and *r* = −0.12 for A1; HIV+: *r* = −0.04 for M1 and *r* = −0.06 for A1, all *p*-values>0.6), thus, age was dropped as a covariate in the following ANCOVA and linear regression analysis. ANCOVA with ethnicity and sex as covariates showed that caliber mean in the A1 segment was significantly greater in HIV+ than in HIV- cohort (*p* = 0.048; Table 1, Figure 2A), but Caliber in the M1 segment had no significant difference between two cohorts (*p* = 0.65; Table 1, Figure 2B). For HIV+ subjects, longer duration of HIV infection was associated with lower A1 caliber (*r* = −0.42, *p* = 0.003, partial correlation coefficient |*r*| = 0.41, Figure 3A). The HIV duration explained 17% of variance in A1 caliber controlling for effect of sex and ethnicity. Higher current CD4+ T cell count also associated with lower A1 caliber (*r* = −0.33, *p* = 0.022; partial correlation coefficient |*r*| = 0.28, Figure 3B). The current CD4+ T cell explained 8% of variance in A1 caliber controlling for effect of sex and ethnicity. An increase in CVD risk was associated with a decrease in A1 caliber in the HIV group (*r* = −0.35, *p* = 0.039, partial correlation coefficient |*r*| = 0.3, Figure 3C), explaining 9.3% of the variance in A1 caliber. There were no significant relationships between A1 caliber and any other markers in either cohort (*p* > 0.05). Furthermore, none of the parameters explored was associated with changes in M1 caliber.

## Discussion

Vascular caliber of the A1-segment of the anterior cerebral artery was found to be higher in patients with HIV infection compared to control subjects when adjusted for sex and race. Interestingly, higher current CD4+ T cell count, longer HIV duration, and increased risk of cardiovascular were associated with a decrease in A1 caliber of HIV+ subjects. These findings are in line with postmortem observations of a larger cross-sectional area of vessels of the Circle of Willis in HIV-infected individuals (2, 3, 5). *In-vivo* measurement of vascular caliber may therefore be an important marker in assessing cerebrovascular effects of HIV infection.

It is possible that this change in lumen caliber could be a reflection of vessel wall thinning or loss of vessel compliance. Indeed, postmortem studies have shown that HIV infection is associated with thinning of the medial arterial layers (2), which has been hypothesized to be a preclinical stage in the development of HIV-related vasculopathy. Studies have observed that adventitial inflammation in HIV predicts a thinner media and leads to dolichoectasia via outward remodeling of arterial lumen (5, 18). It is possible, therefore, that HIV-related pathology causes the smooth muscle in the vessel wall to thin, resulting in outward expansion of the lumen, which was reflected here by an enlarged vessel caliber. It is likely that such phenomenon affects vasculature in the central nervous system, and if so, similar thinning of intima and media would be associated with smaller relative changes to the M1 caliber due to the larger size of that artery, perhaps explaining the lack of observed group-wise and correlational differences. In addition to smooth muscle layer changes, damage to the inner endothelial layer in HIV could affect vascular compliance (7, 19), leading to the luminal enlargement observed here. Remodeling in healthy populations is initiated by detection of signals related to changes in blood flow, wall stress, and shear stress. A functionally intact endothelium can sense changes in these factors and modulate arterial remodeling (20, 21). Following this model, a damaged or dysfunctional endothelial lining could impair the production or passage of vasoconstricting or vasodilating substances, thereby altering the natural course of remodeling (20, 21). Indeed, studies have shown that even in HIV-infected patients on cART, such as the subjects in our study, endothelial dysfunction was present and flow-mediated dilation of arteries was impaired (22–24).

HIV may affect vascular remodeling through the production of toxic proteins. The presence of HIV protein *Tat* was found to cause endothelial dysfunction-dependent non-compliance in coronary arteries (25), and disruption of human brain microvascular endothelial cell functions (26). In a macaque model of Simian Immunodeficiency Virus, the protein *Nef* was shown to cause a wide array of vascular phenotypes ranging from medial hypertrophy to thrombosis of vessels (27). Similarly, in a porcine model of HIV, *Nef* was shown to significantly decrease endothelium-dependent vasorelaxation in pulmonary arteries (28). These studies support our findings of larger A1 caliber in HIV+ compared to HIV- subjects.

Interestingly, the A1 caliber decreased with increasing duration of infection, current CD4+ T-cell levels, and CVD risk in HIV+ subjects, but not in the HIV- controls (when relevant). It is possible that the larger arterial caliber in patients early in the course of HIV infection is a result of arterial remodeling at the acute phase of infection. During the initial period of infection, when the virus is yet uncontrolled by cART, there may be a window of active inflammation where viral proteins and host immune mechanisms are better able to remodel vascular architecture. These results are consistent with a recent study where increased cART duration was found to negatively correlate with retinal caliber, independent of age (6). As the disease progresses despite cART, multiple factors, as discussed below, contribute to increased atherogenesis, which would decrease lumen size. Changes to vascular caliber, for instance, may in part explain why HIV-infected patients have an increased incidence of vascular diseases such as stroke (8). Endothelial dysfunction is considered an event in the progression of atherogenesis (25), and impairment of endothelium-dependent vasorelaxation has been linked to both development and progression of atherosclerosis (23, 29).

Interestingly, cART therapy itself has also been implicated in atherogenic changes, which can lead to an increased cardiovascular risk score (22, 30). The cardiovascular risk score was negatively correlated with A1 caliber in HIV+ subjects, and this reduction in caliber could be an indication of atherogenic changes with time, or a reflection of the inflammatory effects of cART due to immune reconstitution (31). Indeed, cART itself has been shown to cause decreased arteriolar diameter (6, 29). However, studying the effects of cART in our current cohort is limited due to the small sample size at present, and since many patients routinely switch between their cART regimens. A larger, longitudinal study—ideally following patients from the time of diagnosis—may be necessary to parse out the effects of such individual parameters.

A key feature of this study was the effort to use HIV- controls that were socioeconomically matched to the HIV+ population. This enabled us to attempt to control for a variety of extraneous comorbidities and cofactors which may have otherwise confounded results. Analyses of additional vessels in the brain would allow further insight into how widespread this positive arterial remodeling phenomenon is in HIV pathogenesis. A limitation of this study lies in the small number of HIV- subjects (relative to the number of HIV+ participants). Nevertheless, robust differences were seen in the A1 caliber between HIV- and HIV+ cohorts. Additionally, while the HIV- group was younger than the HIV+ subjects, there were no association of any of the clinical parameters with age, in either the univariate or multivariate analysis. The statistics suggest a direct association of A1 caliber with the clinical variables of disease duration, CD4+ T cells, and cardiovascular risk score, with little contribution from age.

The measurement of vascular caliber was performed using novel high-resolution T2^*^-weighted images, and custom software written in-house using Matlab. The T2^*^ images used herein were acquired with 0.55 mm isotropic resolution, resulting in voxels size of 0.17 mm^3^, enabling detection of small changes. This sequence has been shown to be highly efficient way to image brain vasculature, especially after injection of MRI contrast agent (10, 11, 13). The analysis software can also be adapted to obtain measurements from other pulse sequences such as, 3D time-of-flight (TOF) or phase-contrast angiography, or 3D black-blood sequences (32–35). A direct comparison of 3D TOF to 3D T2^*^ weighted images at the same location in the brain (Supplementary Figure 1) depicts the much higher sensitivity of 3D T2^*^ images to detecting blood vessels, which should also translate into ability to detect small changes to vascular caliber. However, these experiments were not systematically performed herein. The detection of vessels using T2^*^ relies on intrinsic magnetic properties of deoxyhemoglobin and MRI contrast agent, and not on physiological and anatomical parameters such as blood flow velocity or direction with respect to imaging slices, making it more uniformly sensitive sequence than traditional angiography methods. In addition, using a segmented EPI sequence enabled imaging of the entire brain in <5 min, and using post-contrast images ensured that the vessels were segmented more reliably by the software, and the cross-sectional area calculated perpendicular to the vessel at each point. Nevertheless, a systematic comparison of the various vascular imaging sequences, and other validatory studies including longitudinal follow-up of patents to confirm these observations need to be performed. In addition, effects of vascular caliber on function can also be explored, e.g., using functional MRI studies. Such studies can help us better understand the findings reported herein.

The novel analysis method described here was able to reliably measure vascular caliber *in-vivo*, where it detected an increased lumen caliber in patients with HIV, and which tended to decrease throughout the course of the illness despite adequate antiretroviral therapy. This suggests that there is extensive remodeling of intracranial vasculature throughout the course of the illness and that these patients may be highly vulnerable to vascular disease. This highly sensitive and reliable method could also be adapted to study vascular complications of other diseases such as diabetes, hypertension, dementia, and inflammatory conditions. Measurements of vascular caliber and detection of arterial remodeling could perhaps be used as *in-vivo* markers of disease progression and may be also used as endpoints in clinical trials of therapeutic candidate drugs.

## Ethics Statement

The data has been acquired after the NIAID Institutional Review Board reviewed and approved the study protocol (clinicaltrials.gov identification number NCT01875588 and NCT00001248; IRB protocol numbers 13-N-0149 and 89-N-0045) and all participants provided written informed consent.

## Author Contributions

GN, DR, and AN: study supervision and concept design. BS, SS, GN, and AN: patient recruitment and acquisition of data. PD, CM, C-YL, SR, JS, ET, DR, GN, and AN: drafting and revising of manuscript. PD, TW, CA, DR, GN, and AN: data analysis including statistical analysis and interpretation. BS, CM, C-YL, SR, ET, DR, and AN: obtaining funding.

### Conflict of Interest Statement

The authors declare that the research was conducted in the absence of any commercial or financial relationships that could be construed as a potential conflict of interest.

## Supplementary Material

Supplementary Figure 1 Comparison of intracranial vasculature by 3D-T2^*^ (on the left, vessels are dark, and some are marked by arrows on one of the hemispheres for clarity) and Time Of Flight (TOF, on the right, vessels are bright) on an additional subject. Both scans were acquired at similar resolutions after injection gadobutrol.
