# Supplementary figures and images for: In-vivo MRI Reveals Changes to Intracerebral Vasculature Caliber in HIV Infection
Source: Front Neurol. 2019 Jun 26;10:687. doi: 10.3389/fneur.2019.00687 (PMC6607694; doi:10.3389/fneur.2019.00687)

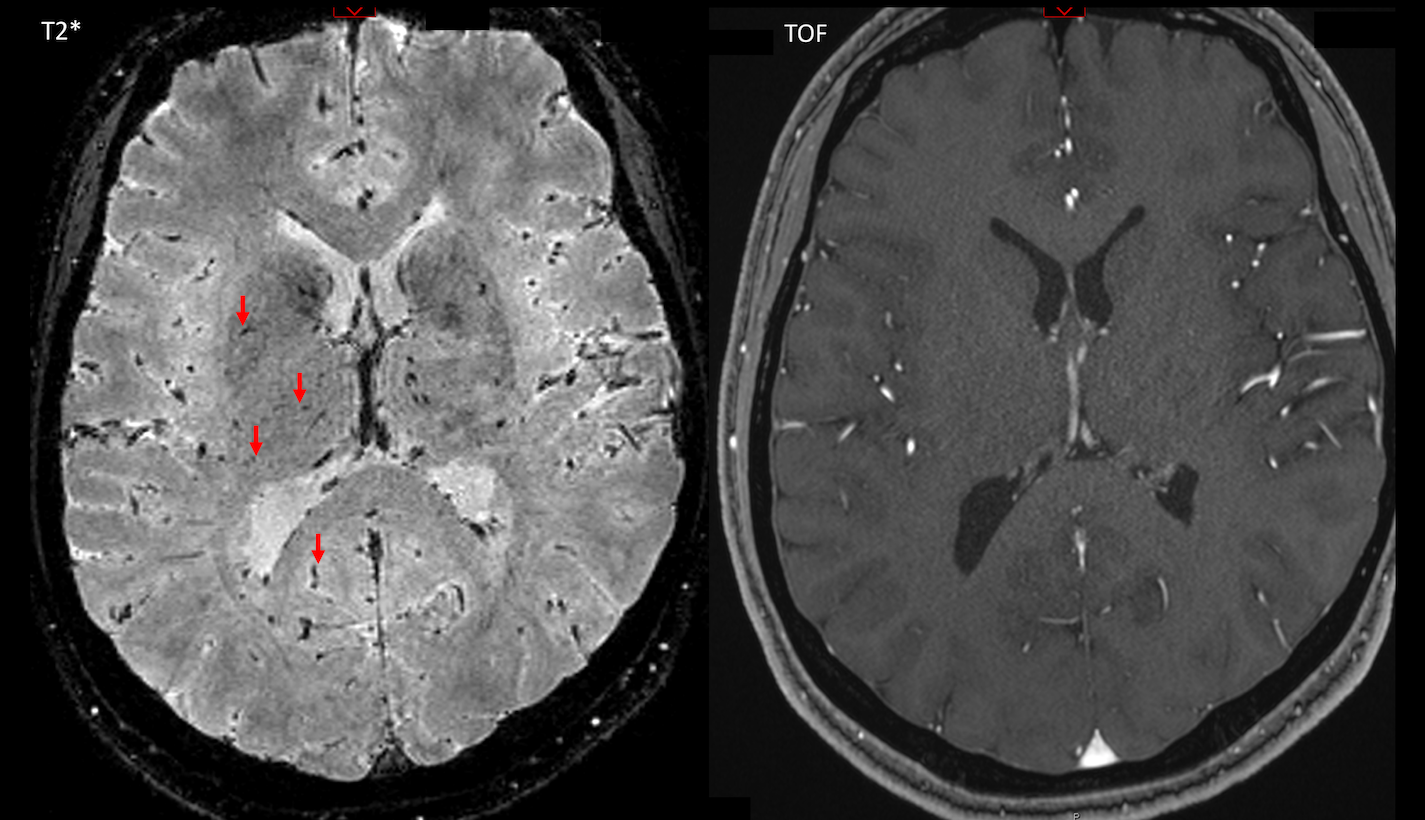

Supplement: Supplementary Figure 1 — Comparison of intracranial vasculature by 3D-T2* (on the left, vessels are dark, and some are marked by arrows on one of the hemispheres for clarity) and Time Of Flight (TOF, on the right, vessels are bright) on an additional subject. Both scans were acquired at similar resolutions after injection gadobutrol. [file Image_1.tiff]
